# The C-terminus of *S. pombe* DDK subunit Dfp1 is required for meiosis-specific transcription and cohesin cleavage

**DOI:** 10.1242/bio.20135173

**Published:** 2013-06-11

**Authors:** Anh-Huy Le, Tara L. Mastro, Susan L. Forsburg

**Affiliations:** Program in Molecular and Computational Biology, University of Southern California, Los Angeles, CA 90089-2910, USA

**Keywords:** Cdc7, Hsk1, Dbf4, Meiosis, Fission yeast, Rec8

## Abstract

The DDK complex is a conserved kinase complex, consisting of a catalytic subunit, Hsk1 (Cdc7), and its regulatory subunit Dfp1 (Dbf4). This kinase is essential for DNA replication. In this work, we show that *dfp1-r35*, which truncates the Dfp1 C-terminus zinc finger, causes severe meiotic defects, including reduced spore viability, reduced formation of programmed double strand breaks, altered expression of meiotic genes, and disrupted chromosome segregation. There is a high frequency of dyad formation. Mutants are also defective in the phosphorylation and degradation of the meiotic cohesion, Rec8, resulting in a failure to proceed through the MII division. These defects are more pronounced in a haploid meiosis model than in a normal diploid meiosis. Thus, several critical meiotic functions are linked specifically to the C-terminus of Dfp1, which may target specific substrates for phosphorylation by Hsk1.

## Introduction

The *S. pombe* DDK (Dbf4-Dependent Kinase) complex is a conserved, essential kinase consisting of a catalytic subunit Hsk1 (Cdc7 in humans or budding yeast) and its regulatory subunit Dfp1 (Dbf4 in humans or budding yeast) (reviewed by Duncker and Brown, 2003; Kim et al., 2003; Labib, 2010; Sclafani, 2000). In vegetative cells, DDK has an essential function in the initiation of DNA replication as well as DNA repair and checkpoint activities (reviewed by Duncker and Brown, 2003; Kim et al., 2003; Labib, 2010; Sclafani, 2000). *S. pombe dfp1^+^* is regulated transcriptionally and post-transcriptionally to restrict kinase activity to S phase (Brown and Kelly, 1999; Takeda et al., 1999; Tatebe et al., 2001). Dbf4 and its orthologues contain several well conserved domains to which different functions have been linked (Bailis and Forsburg, 2004; Dolan et al., 2010; Duncker et al., 2002; Fung et al., 2002; Gabrielse et al., 2006; Harkins et al., 2009; Takeda et al., 1999; Varrin et al., 2005). These include an N-terminal domain which is required for checkpoint response, a middle domain M which is associated with replication activities, the short MIR motif which is required for association with the Swi6 heterochromatin protein contributing to sister chromatid cohesion and timely replication in the centromere, and the extreme C-terminal domain which is required for normal response to alkylation damage and required for meiosis. A simple model suggests that these different domains of Dfp1 target the DDK to different substrates, although the C-terminal mutations are also associated with reduced kinase activity (Fung et al., 2002; Harkins et al., 2009).

DDK has been linked to multiple roles throughout meiosis. In *S. cerevisiae*, early studies on a temperature sensitive allele of CDC7 showed the kinase is required for meiotic recombination (Buck et al., 1991; Sclafani et al., 1988). More recent experiments have used selectively timed inactivation to demonstrate that DDK complex is required for meiotic replication initiation (Valentin et al., 2006; Wan et al., 2006), but also functions in recombination (Lo et al., 2008; Matos et al., 2008; Ogino and Masai, 2006; Wan et al., 2008; Wan et al., 2006). In *S. cerevisae* and in *S. pombe*, DDK is required to recruit the nuclease SpRec12/ScSpo11 to the chromosome to generate programmed double strand breaks (prDSBs) (Ogino et al., 2006; Sasanuma et al., 2008; Wan et al., 2008; Wan et al., 2006). The meiotic transcriptional program is also regulated by Cdc7 (Lo et al., 2012). *S. cerevisiae cdc7* mutants that complete meiosis often produce diploid dyads, which is linked to the failure of monopolar attachment at the kinetochore (Lo et al., 2008; Matos et al., 2008; Rabitsch et al., 2003; Tóth et al., 2000). Loss of Rec8 has been shown to relieve the anaphase I arrest in DDK-depleted cells after meiotic S phase (meiS) (Valentin et al., 2006). Recently, *S. cerevisae* Cdc7 and CK1 kinases were found to independently collaborate in the degradation of Rec8 (Ishiguro et al., 2010; Katis et al., 2010).

In this work, we make use of a previously isolated *S. pombe dfp1^+^* allele lacking the C-terminus (*dfp1-(1-519)*, also known as *rad35*) to study the role of DDK in meiosis. For simplicity, we will refer to this allele as *dfp1-r35*. Earlier, we showed that this is a separation-of-function allele that shows a subset of DDK-associated phenotypes; *dfp1-r35* cells are proficient for replication, but defective in response to alkylation damage (Dolan et al., 2010). Here, we show that *dfp1-r35* cells are proficient for meiS phase, but show subsequent defects in the meiotic program including reduced spore viability, reduced prDSBs, delayed expression of meiotic genes, and disrupted chromosome segregation. There is a high frequency of dyad formation. Mutants are also defective in the phosphorylation and degradation of the meiotic cohesin Rec8, resulting in a failure to proceed through the MII division. Thus, several critical meiotic functions are linked specifically to the C-terminal zinc-finger of Dfp1, suggesting it targets the Hsk1 kinase to several different meiosis-specific substrates. Finally, we demonstrate that the meiotic phenotype of *dfp1-r35* varies depending on the presence of homologous chromosomes.

## Materials and Methods

### Strains and media

*S. pombe* strains are indicated in supplementary material Table S1. Standard genetic techniques and media were used to construct and maintain strains (Sabatinos and Forsburg, 2010).

### Microscopy

For terminal meiotic phenotypes, microscopy was performed on live or ethanol fixed cells stained with DAPI (Sabatinos and Forsburg, 2010) using a 63× oil-immersion lens (PLApo, NA = 1.32) on a Leica DMR fluorescence microscope. Images were collected with Openlab 3.6.1 contrast adjusted and assembled in Canvas 12.

For LacI-GFP chomosome segregation live cell imaging, cells were plated on agarose pads as described previously (Green et al., 2009). Images were acquired with a DeltaVision Core wide field deconvolution microscope (Applied Precision, Issaquah, WA) using an Olympus 60×/1.40, PlanApo, NA = 1.40 objective lens and a 12-bit Photometrics CoolSnap HQII CCD, deep-cooled, Sony ICX-285 chip. The system x–y pixel size is 0.1092 µm x–y. softWoRx v4.1 (Applied Precision, Issaquah, WA) software was used at acquisition electronic gain = 1.0 and pixel binning 1×1. Excitation illumination was from a Solid-state illuminator (7 color version), GFP was excited and detected with a (ex)475/28,(em)525/50 filter set and a 0.2 second exposure. A polychroic mirror was used GFP/mCherry Chroma ET C125705 roughly: 520/50–630/80. Nine z sections at 0.5 µm were acquired. 3-D stacks were deconvolved with manufacturer provided OTFs using a constrained iterative algorithm and images were maximum intensity projected. Images were contrast adjusted using a histogram stretch with an equivalent scale and gamma for comparability. Brightfield images were acquired with DIC. Images were assembled using softWoRx Explorer v. 1.3.0 and Photoshop CS3 v. 10.0.1.

### Spore viability assay and recombination assays

The recombination assay was performed as described previously (Catlett and Forsburg, 2003). Indicated strains were mated on ME agar plates for three days at 25°C. Asci were treated with 0.5% glusulase (Perkin Elmer) solution and incubated on a rotating platform overnight at room temperature. For each trial spores for all strains were distributed over ten plates. For each trial, approximately 5,000, 35,000, or 100,000 spores were plated for wild type, *rec12*Δ, or *dfp1-r35* respectively. Spore viability is represented as the fraction of resulting colonies over the total number of plated spores. The standard deviation was calculated from four trials. Spore viability of mutants was normalized to wild type, which was defined as 100% viable. YES plates were replica plated onto EMM plates lacking histidine, lysine, adenine, or histidine and lysine. The fraction of autotrophs on the respective media was tabulated to determine recombination frequencies. Diploids were distinguished from haploids via cell morphology, color on phloxinB, and FACs. Data were pooled from four trials.

### Induction of meiosis

For homothallic *h90* strains, cultures were grown overnight at 25°C to an OD_595 nm_ of 0.8 in 10 ml of EMM media with appropriate supplements. Cells were washed twice in 10 ml EMM-N media, and then starved in ME media for 30 hrs. 1 ml of cells suspension was fixed in 70% ethanol, and stained with DAPI (Sabatinos and Forsburg, 2010).

Induction of meiosis for live cell imaging was accomplished by co-culturing *h^−^* and *h^+^* strains of appropriate genotype independently at 32°C to OD of ∼1. Cells were then washed 2× in EMM-N. Cell pellets of each were combined into 12 ml ME media at 25°C for 12–16 hrs. Cells were then centrifuged at low speed (1000 *g*) and placed on a SPAS 2% agarose pad for imaging at 25°C.

Induction of synchronous meiosis was carried out using a *pat1* temperature sensitive allele as described previously (Forsburg and Hodson, 2000). Briefly, 200 ml of cell cultures were grown in EMM media supplemented with appropriate nutritional supplements overnight to reach an OD_595 nm_ of ∼0.8. Cultures were washed twice in EMM-N media and were starved at 25°C in 200 ml of EMM-N Media for ∼17 hrs in order to arrest cells in G1. Haploid strains were supplemented with 7 mg/ml adenine. To induce meiosis in cell cultures, the temperature was shifted to 34°C by adding an equal volume of pre-heated (36°C) EMM media containing 1 g/l of NH4Cl and 70 mg/ml uracil, adenine and leucine to starved cultures, and by incubating cultures in a water bath at 34°C. Samples for nuclear counts, FACS, RT-PCR, and Westerns were taken at indicated times.

### Flow cytometry (FACS)

Flow cytometry was performed as described previously (Sabatinos and Forsburg, 2009). Briefly, 200 ml of cell suspension fixed in 70% ethanol were rehydrated by washing twice in 1 ml of 0.05 M sodium citrate solution and removing the supernatant after each wash. Rehydrated cells were resuspended in 500 ml of 0.05 M sodium citrate solution with 0.1 mg/ml RNaseA, and incubated for 90 minutes at 36°C. Cells were stained with Sytox Green (Invitrogen) by adding 500 ml 0.05 M sodium citrate solution with 0.1 mM Sytox Green. Cell suspensions were sonicated with three pulses at 20% amplitude, and vortexed once more shortly before submission of samples to FACS analysis.

### Reverse transcriptase PCR

RNA was extracted using QIAGEN's RNAse Easy RNA extraction kit. Extracted RNA was quantified on a spectrophotometer (Nanodrop, ND1000). RNA was converted to cDNA using Roche kit. Relative transcription levels were determined by performing multiplex PCR using target primer and actin primers as described above (supplementary material Table S2). Actin primer sequences from Kloc et al. were used (Kloc et al., 2008). Quantification was done using QuantOne software version 4.6.9 taking a ratio of target to actin signal.

### Detection of meiotic prDSBs by pulsed field gel electrophoresis (PFGE)

Agarose plugs were prepared as described previously (Cervantes et al., 2000). Briefly, 50 ml of cultures were stopped by adding 500 ml of 20% sodium azide solution to the culture and incubating the culture on ice for 5 minutes. Harvested cultures were spun down, washed once in 10 ml of PBS buffer, and once in 10 ml of CSE buffer (20 mM citric acid, 20 mM Na_2_HPO_4_, 40 mM EDTA, 1.2 M sorbitol at pH 5.6). Each culture was digested in CSE media containing 0.45 mg/ml of Lysing Enzyme, and 0.02 mg/ml of zymo 100T for 20–40 minutes. Digestion was monitored by microscopy. Plugs were prepared from digested cell pellets that were resuspended in TSE (10 mM Tris pH 7.5, 45 mM EDTA, pH 8.0, 0.9 M sorbitol) to make a cell suspension of 3.0×10^9^ cells/ml. Plugs were treated with Proteinase K in Sarkosyl-EDTA (1% sarkosyl, 0.5 M EDTA, 1 mg/ml Proteinase K, calibrated to a pH of 9.5) solution for 48 hours. The Sarkosyl-EDTA-Proteinase K solution was changed once after 24 hours. Plugs were washed three times in 10 ml TE Buffer, and three times in 10 ml TAE buffer (40 mM Tris acetate, 1 mM EDTA at pH 8.0).

PFGEs were run on a Biorad Chef II Pulse Field machine with the following specifications: 48 hours at 2 Volts/cm at an angle of 106° and pulse times ramping from 1200 to 1800 seconds. The gel was stained in 200 ml of TAE Buffer containing SYBR Green for 20 minutes, and bands were visualized in a ChemiDoc Machine (XRS, BioRad). Quantification of breaks using the ratio of breaks/chromosome signal, as described previously (Borde et al., 2000a; Borde et al., 2000b), with BioRad QuantOne software version 4.6.9.

### Determination of Rec8 stability

We performed video microscopy on diploid cells entering meiosis expressing Rec8-GFP and quantified these by scoring the time at which the Rec8-GFP signal decreased from pan-nuclear to a focus in relation to the MI division. The time at which the Rec8-GFP focus disappeared was measured from the point at which the pan-nuclear signal became reduced. A two-tailed t-test was applied to determine significance in the different strains.

For analysis of protein, TCA precipitation was performed as described previously (Foiani et al., 1994). Cell cultures were stopped by adding 10× STOP buffer containing sodium azide solution to harvested culture and incubating the cultures on ice for 10 minutes. Cells were washed in PBS buffer (137 mM NaCl, 2.7 mM KCl, 4.3 mM Na_2_HPO_4_, 1.47 mM KH_2_PO_4_) and then MQ water. Protein was extracted using TCA precipitation. Protein extracts were quantified using BCA and an equal amount of protein for each sample was run on a 8% SDS PAGE gel containing 1.25% crosslinker. Membrane was blocked in 5% PBST (1× PBS solution with 2% Tween-20). To detect Rec8-GFP, membrane was incubated in 5% PBST milk containing a 1:2000 dilution of JL8 monoclonal antibodies (Clontech) overnight at 4°C, and washed three times for 10 minutes in 10 ml PBST each time. Blots were incubated in 5% PBST milk solution with 1:3000 secondary goat anti-mouse HRP antibodies (Millipore). Blots were developed using ECL (Pierce). Quantification was done using QuantOne software version 4.6.9 taking a ratio of GFP to PCNA signal.

## Results

### Defective meiosis in *dfp1-r35* cells

**Fig. 1.**
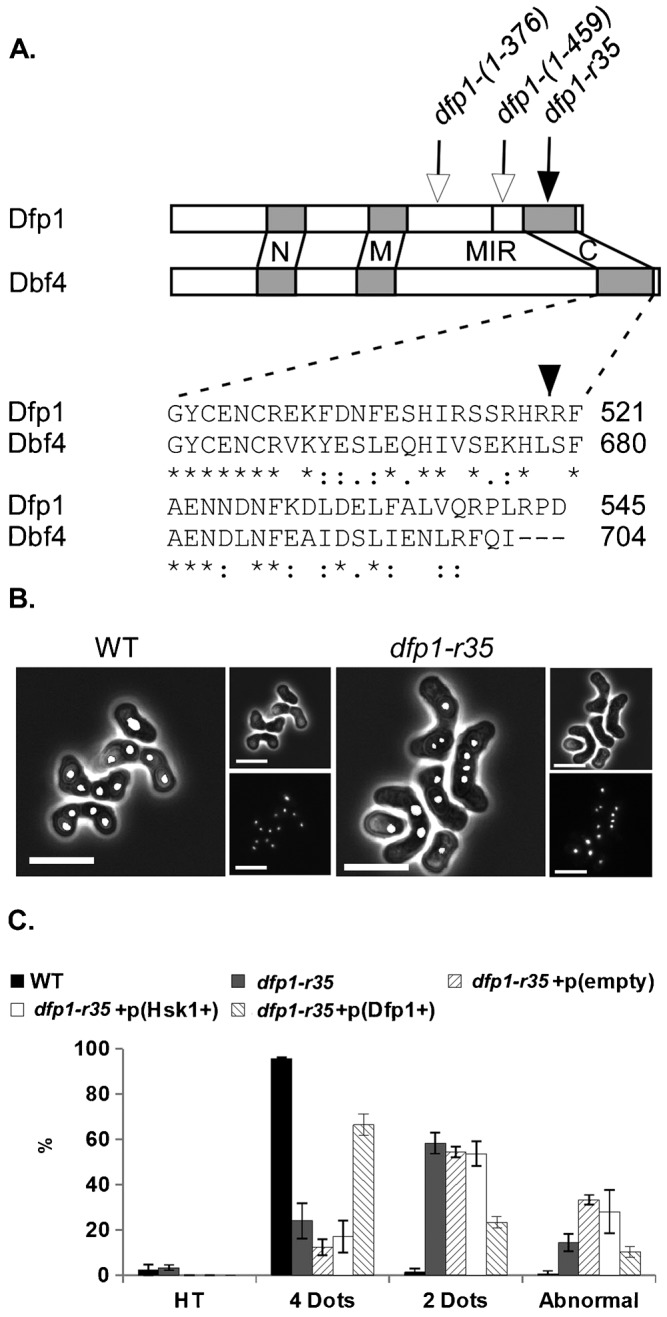
Terminal meiotic phenotype of *dfp1-r35* mutants. (**A**) Structure of Dfp1. *dfp1-r35* mutation is a truncation within a zinc-finger domain that is conserved between *S. pombe* and *S. cerevisiae*. (**B**) Nuclear morphology of terminal meiotic products of *dfp1-r35* (FY1154) compared to WT (FY155) visualized with DAPI staining. Scale bar: 10 mm. (**C**) Quantification of terminal meiotic phenotypes as horsetailing (HT), 2 DAPI stained bodies, 4 DAPI stained bodies, or abnormal. Defects of *dfp1-r35* are suppressed by ectopically expressed *dfp1*^+^. 300 asci for each strain were analyzed and error bars represent standard deviation.

The Dfp1 protein is an essential regulatory subunit for the Hsk1 (Cdc7) kinase, and targets the kinase to different substrates (reviewed by Duncker and Brown, 2003; Labib, 2010). The C-terminus of *S. pombe* Dfp1 contains a putative zinc finger similar in sequence to the C-terminus of *S. cerevisiae* Dbf4 (Fig. 1A). Several truncations of this C-terminus have been constructed; the mutants are viable and therefore competent for S phase and the replication function of DDK, but they are defective in response to alkylating damage (Dolan et al., 2010; Fung et al., 2002; Ogino et al., 2001). Thus, they function similar to separation of function alleles. The *dfp1-r35* mutant, which truncates just 21 amino acids from the C-terminus of the Dfp1 protein (Fig. 1A), was previously reported to have defects in the response to alkylation damage (Dolan et al., 2010).

Wild type homothallic (*h90*) cells undergoing meiosis produce asci with four regularly shaped spores and evenly segregated nuclei (Fig. 1B). However, *h90 dfp1-r35* cells form asci with aberrant morphologies (Fig. 1B). Only about 24% of observed asci produce four spores. The majority (58%) produce two spored-asci. In about 14% of asci, at least one spore contains fragmented or multiple nuclei (Fig. 1C). Generally, spores are of different sizes, and spore viability is significantly reduced (1.81±1.46%) even when compared to *rec12*Δ mutants (20.99±5.17%) that fail to induce prDSBs (supplementary material Table S3). The aberrant ascus morphologies of *dfp1-r35* are rescued by a plasmid containing *dfp1*^+^, but not by a plasmid containing *hsk1*^+^, indicating that the observed effects are specific to the absence of the Dfp1 C-terminus (Fig. 1C).

In order to test how *dfp1-r35* affects the progression of meiotic S phase (meiS phase), we used the temperature sensitive *pat1-114* mutation to induce a synchronous meiosis. Pat1 kinase is an inhibitor of meiosis, and shifting the temperature to 34°C will cause the mutant to enter meiosis from either a haploid or a diploid state. While the haploid is frequently used as a meiotic model, the absence of homologous chromosomes and mating type heterozygosity leads to some differences in meiotic dynamics (Pankratz and Forsburg, 2005; Yamamoto and Hiraoka, 2003). Therefore, we constructed a stable *h^−^/mat2-102 pat1-114/pat1-114* diploid that maintain heterozygosity and diploidy, and can only enter meiosis when Pat1 is inactivated. This allows synchronous meiotic progression as in Pankratz and Forsburg (Pankratz and Forsburg, 2005). We compared the effects of *dfp1-r35* to wild type and *rec12*Δ in homozygous *h^−^/mat2-102* diploids.

**Fig. 2.**
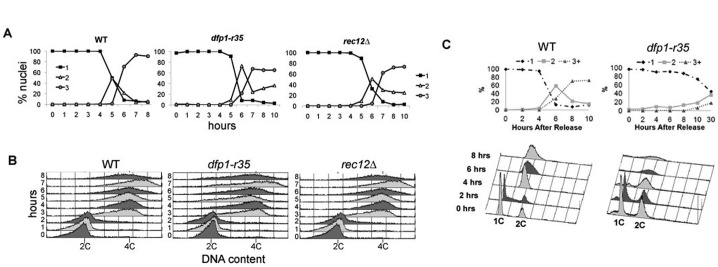
Synchronous meiosis in *mat2-102/h^−^ pat1-114/pat1-114* diploids and *pat1-114* haploids. (**A**) Comparison of DAPI stained profiles categorizing 1, 2, or ≥3 DAPI stained bodies for WT, *dfp1-r35*, and *rec12*Δ following meiotic induction using *pat1-114*. (**B**) FACS profiles demonstrating the progression of meiotic replication through meiotic induction. DNA content moves from 2C to 4C. (**C**) Comparison of haploid meiotic induction of *dfp1-r35* compared to wild type. Both FACs and DAPI staining was done as in panels A and B to monitor meiotic progression. Strains: wild type (FY6332/FY6336), *dfp1-r35* (FY6347/FY6378), *rec12*Δ (FY6530/FY6531), wild type (FY4129), *dfp1-r35* (FY4396).

As expected, wild type and *rec12*Δ mutants enter and complete meiotic S phase between two and three hours. There was no evident delay in meiotic DNA replication in *dfp1-r35* mutants following induction of meiosis (Fig. 2B), consistent with our prior observations in vegetative cells (Dolan et al., 2010). We monitored progression through meiotic divisions by counting the number of nuclei, where the MI division yields 2 DAPI staining spots and the MII division yields ≥3. Over the time course of this experiment, wild type strains showed MI occurring at 5 hours and MII at 7 hours with nearly all cells completing both divisions. In contrast, approximately 25% of *dfp1-r35 pat1* mutants did not complete the MII division. There was also a 1 hour delay in the MI division (Fig. 2A). We observed normal MI dynamics in *rec12*Δ mutants, but a reduced efficiency of MII, similar to previous reports (Fig. 2A) (Davis and Smith, 2003). However, this defect was not as severe as *dfp1-r35*.

We compared these results to the same experiment in *dfp1-r35 pat1-114* haploids, in which cells are induced to undergo meiosis in the absence of homologous chromosomes. These also proceeded normally through meiotic S phase, but showed a more striking delay of MI. There was no evidence for MII even after 30 hours (Fig. 2C). Thus, the haploid *pat1*-induced meiosis is more sensitive to *dfp1-r35* mutation than the diploid, and in both conditions, the defect appears to occur after meiS phase.

### *dfp1-r35* disrupts meiotic transcription

**Fig. 3.**
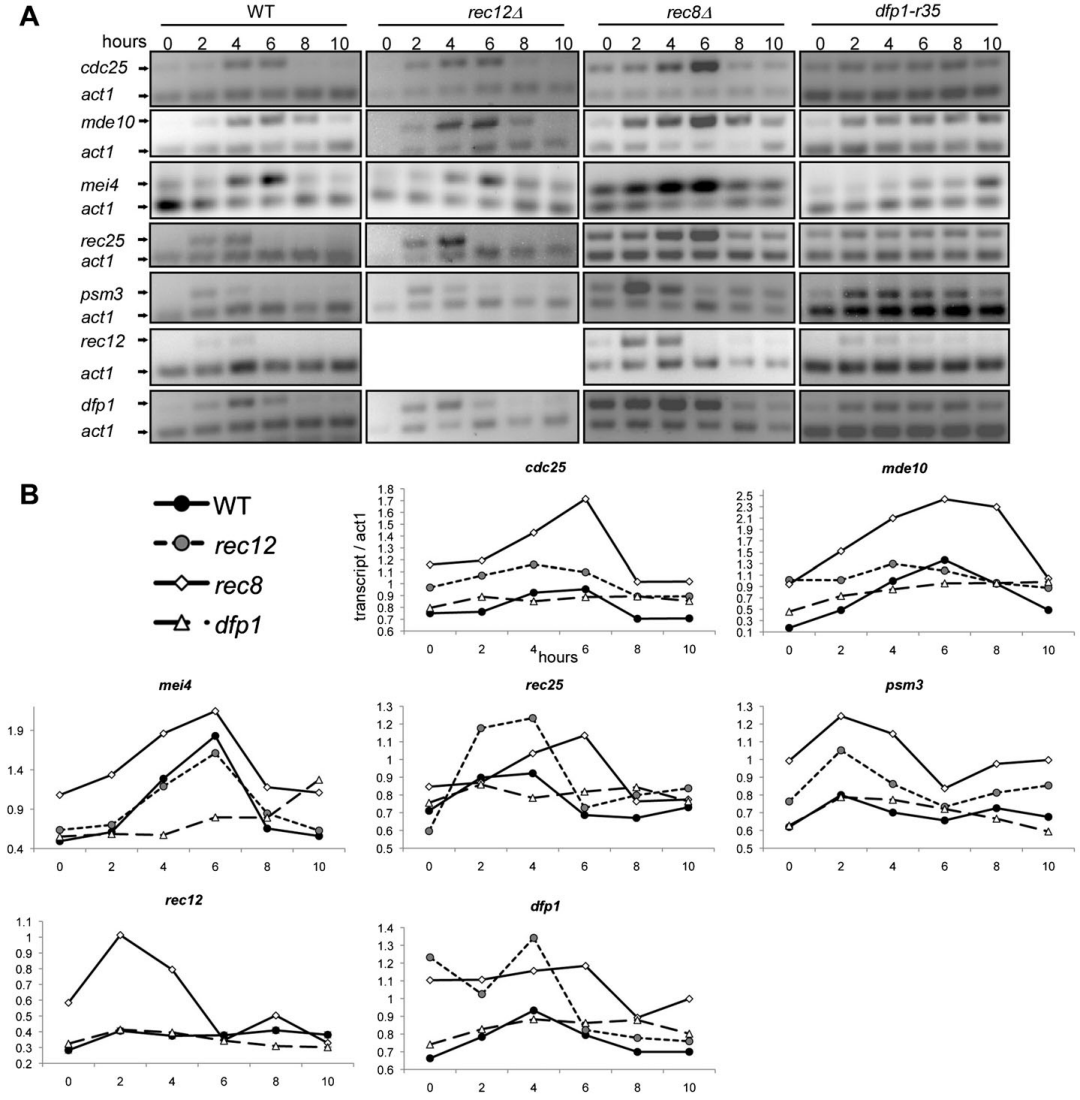
Transcriptional expression of meiotic genes. RT-PCR of *pat1-114* (FY4129), *pat1-114 rec12Δ* (FY2008), *pat1-114 rec8Δ* (FY1955) and *pat1-114 dfp1-r35* (FY4396) cells undergoing synchronous haploid meiosis shows expression with altered timing of meiotic markers: mid/late transcripts (*cdc25^+^*, *mde10^+^*, *mei4^+^*), early rep1-independent (*rec25*^+^) and -dependent (*psm3^+^*, *rec12^+^*, *dfp1^+^*). (**A**) Visualization of RT-PCR using SYTOX green on agarose gel. (**B**) Quantification and graphical representation of panel A.

The delay in meiosis in *dfp-r35* haploid mutants could reflect a delay in the overall meiotic program. We examined the timing of meiotic transcript accumulation, which occurs in characteristic waves (Mata et al., 2007). Early meiotic gene expression depends in part on the meiosis-specific DNA synthesis control-like transcription factor complex (DSC1) (Cunliffe et al., 2004; Mata et al., 2007), which regulates *rec12^+^*, *dfp1^+^*, and numerous other genes. There is also DSC1-independent early transcription of several genes including *rec25*^+^ and *rec27*^+^ (Mata et al., 2007). The middle wave depends on expression of *mei4*^+^, a transcription factor that itself regulates later gene expression including *cdc25*^+^ and *mde10*^+^ (Horie et al., 1998; Murakami-Tonami et al., 2007; Nakamura et al., 2004). Transcript accumulation is also affected by the presence of regulatory sequences called DSR elements, which target the mRNA for turnover. These are found on a number of genes including *rec8*^+^ and *mei4*^+^ and prevent premature accumulation of their transcripts (Yamamoto, 2010). We investigated progression through the meiotic program by monitoring accumulation of messages from several meiosis-specific genes: the DSC targets *psm3*^+^, *rec12*^+^ and *dfp1*^+^, a non-DSC early gene *rec25*^+^, middle meiotic genes *mei4*^+^, and *mei4*^+^-dependent transcripts *cdc25*^+^ and *mde10*^+^ (Fig. 3).

In *pat1-114* control cells, *psm3*^+^ and *rec12*^+^ message levels peaked at 2 hours, quickly decreased again by 6 hours, and showed a slight increase again by 8 hours. Transcription of both *mei4*^+^ and *mde10*^+^ peaked at 6 hours. Expression of *cdc25*^+^, another *mei4*^+^-target, peaked between 4 and 6 hours. This is consistent with previous descriptions of meiotic gene expression (Mata et al., 2002; Mata et al., 2007).

These patterns were significantly changed in *dfp1-r35* mutants. Both *psm3*^+^ and *rec12*^+^ messages were induced with normal timing but decreased at a slower rate and did not experience a rebound at 8 hours. Transcription of *mei4*^+^, *mde10*^+^, *cdc25*^+^ and failed to cycle as in wild type; however, levels of *mei4*^+^ and *mde10*^+^ increased throughout the time course. Additionally, *dfp1*^+^ transcription itself was altered, as it did not decrease within the time course. Thus, while meiosis-specific gene expression occurs in *dfp1-r35* cells, it is significantly disrupted relative to the normal program.

We compared these results to transcription patterns in *rec8*Δ, which lacks the meiotic cohesin and *rec12*Δ which lacks the ability to make DSBs (Cervantes et al., 2000; Parisi et al., 1999; Watanabe and Nurse, 1999). Expression of the meiosis-specific transcripts was not affected in *rec12*Δ, despite the lack of prDSBs, indicating that the transcription program is largely independent of Rec12 or DSB formation (Fig. 3). In *rec8*Δ *pat1-114* mutants, we observed that the peak of all but *rec25*^+^ occurred similar to wild type; however, the levels were noticeably higher. Interestingly for both *rec8*Δ and *rec12*Δ, *dfp1*^+^ levels were elevated compared to wild type and were already increased upon meiotic induction. Thus, both *rec8*Δ and *dfp1-r35* cause dysregulation of the meiotic transcriptional program, although the patterns are different. Importantly, the expression of meiosis-specific transcripts also confirms that the *dfp1-r35* cells have entered meiosis.

### Recombination and induction of prDSBs in Dfp1 C-terminal truncation mutants

Previous studies have shown that DDK is required for recombination and induction of prDSBs in both budding and fission yeast (Ogino et al., 2006; Sasanuma et al., 2008; Wan et al., 2008; Wan et al., 2006). We examined recombination in known genetic intervals in *dfp1-r35* relative to wild type diploids. Intergenic recombination was determined in the interval between *lys4-95* and *his4-239*, located on chromosome II, and intragenic recombination in the *ade6* hotspot was measured using the *ade6-52* and *ade6-26* point mutations located on chromosome III (supplementary material Table S4) (Ponticelli et al., 1988). In wild type cells, 3.98% of germinating spores were both His^+^ and Lys^+^ recombinants. As expected, except for one colony, all the His^+^ Lys^+^ spores recovered from *rec12Δ* were diploids. In *dfp1-r35* mutants, a high fraction of diploids was also recovered. In the few haploids, a sharp reduction in intergenic recombination was observed; however unlike *rec12Δ*, it was still detectable at 0.108% His^+^ Lys^+^. We observed a similar reduction in intragenic recombination; in wild type cells, about 0.285% of germinating spores were Ade^+^. For *rec12*Δ, we recovered no Ade^+^ cells, and for *dfp1-r35* mutants we recovered one colony, for a rate of 0.007%.

**Fig. 4.**
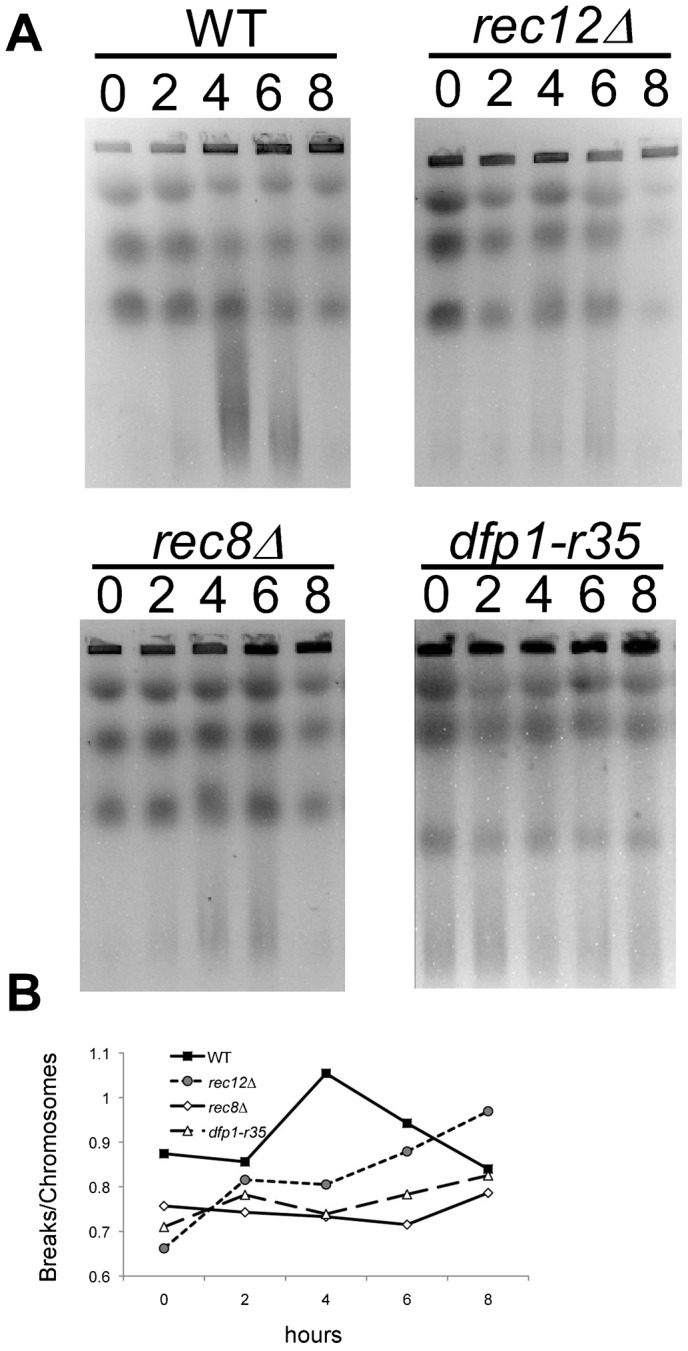
prDSBs in *dfp1-r35* mutants. (**A**) Pulsed field gel electrophoresis (PFGE) of WT (FY4129), *rec12*Δ (FY2008), *rec8*Δ (FY1955) or *dfp1-r35* (FY4396) *pat1-114* cells undergoing synchronous meiosis. *dfp1-r35* mutants, similar to *rec12*Δ mutants, cannot induce prDSBs. (**B**) Quantification of PFGE in panel A using the ratio of breaks/chromosome signal as previously described by Borde et al. (Borde et al., 2000a).

*hsk1* mutants are defective in the induction of prDSBs because they disrupt the localization of the endonuclease Rec12 (Green et al., 2009; Sasanuma et al., 2008; Wan et al., 2008). To test whether the recombination defect in *dfp1-r35* was due to the inability to induce prDSBs, we performed pulsed field gel analysis on agarose plugs prepared from cell cultures undergoing synchronous pat1 meiosis (Fig. 4B). Wild type cells typically exhibit a smear below chromosome III that is observed between 2–4 hours, which has been shown to represent prDSBs. We observed that prDSBs are significantly reduced or absent in the negative control *strains pat1-114 rec8*Δ, and *pat1 rec12*Δ mutants, consistent with previous observations (Cervantes et al., 2000; Ogino et al., 2006). We found that *pat1-114 dfp1-r35* mutants exhibit a smear in all time points; this is consistent with a constitutive level of DNA damage and DNA breaks, which was also observed in vegetative cells (Dolan et al., 2010). However, the smear does not increase in intensity as in wild type, suggesting that the C-terminus of Dfp1 is important for inducing prDSBs during meiosis.

### Chromosome segregation defects in Dfp1 C-terminal truncation mutants

Importantly, the meiotic phenotype of *dfp1-r35* suggests that disruption in prDSB formation is not its only defect, because *rec12*Δ mutants, which are completely defective for prDSB formation, nevertheless complete both meiotic divisions in with a normal timing and transcriptional program. Though *rec12*Δ mutants also form dyads (Fig. 2A) (Davis and Smith, 2003), the level of dyad formation of the *dfp1-35* mutant is dramatically higher than that in the *rec12*Δ cells. Consistent with previous findings (Davis and Smith, 2003), *rec12*Δ spores are about 20% viable. In contrast, *dfp1-r35* mutants retain only about 2% spore viability. The loss of viability as well as the disruption in segregation and spore formation suggests that Dfp1 contributes to additional activities, independent of Rec12-driven prDSBs.

Because *dfp1* mutants have a high fraction of dyad asci, we asked whether this division resembles MI (reductional) or MII (equational) divisions, by analyzing sister chromatid segregation. We constructed diploid strains heterozygous for lacO binding sites at the centromere-linked *lys1*^+^ marker (Tatebe et al., 2001). Upon expression of a lacI-GFP reporter, this array can be visualized via a single GFP focus associated with one of the chromosome I homologues.

**Fig. 5.**
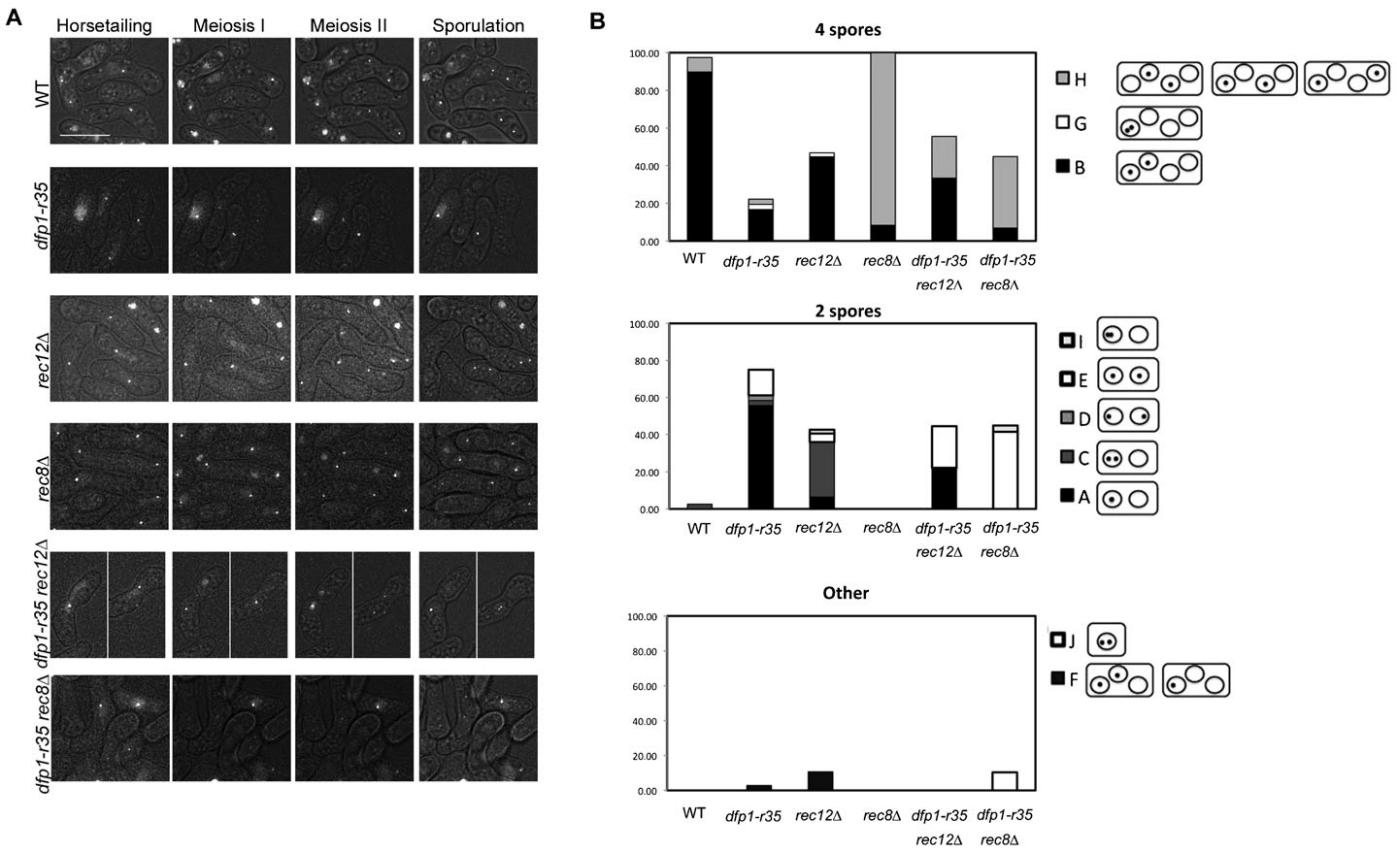
Chromosome segregation in *dfp1-r35* mutants compared to *rec8Δ* and *rec12Δ*. (**A**) Representative images from live cell analysis of LacI-GFP chromosome segregation assay in diploid cells (supplementary material Movies 1–6). The indicated strains heterozygous for lys1^+^::lacO lacI-GFP adjacent to centromere I were grown at (32°C) and plated on agarose pads for image acquisition (see Materials and Methods). Strains: wild type (FY6221×FY6331), *rec8*Δ (FYFY5916×FY6131), *rec12*Δ (FY5197×FY6134), *dfp1-r35* (FY6236×FY6441), *dfp1-r35 rec8*Δ (FY6204×FY6416), *dfp1-r35 rec12*Δ (FY6143×FY6175). Scale bar: 10 µm. Selected panels display predominate segregation phenotype for each mutant observed. (**B**) Graphical representation of quantification of live cell imaging classes. See supplementary material Table S6 for class descriptions and quantities.

Using live cell imaging, we monitored meiosis and spore formation. In wild type diploids, we observed that 97.44% of the meiotic events produced a four spore ascus (Fig. 5; supplementary material Table S5; Movies 1–6). 92.31% of wild type showed the expected reductional segregation at MI, in which the lacI-GFP signal remains in one nucleus (Fig. 5; supplementary material Table S5). During the reductional MII division, the lacI-GFP signal separates, and because fission yeast tetrads are ordered, this results in two adjacent spores containing the signal (Yokobayashi et al., 2003).

In contrast, while *rec8*Δ mutants also mostly formed four spores, in 91.67% of cells, the MI division was equational and the lacI-GFP signal split (Fig. 5; supplementary material Table S5). This is similar to the data reported from terminal phenotype analysis for *rec8*Δ mutants (Molnar et al., 2001; Yokobayashi et al., 2003).

Both *dfp1-r35* and *rec12*Δ diploids exhibited a variety of aberrant chromosome segregation phenotypes in live analysis. Both *dfp1-r35* (75%) and *rec12*Δ (43%) formed dyads at a high rate. Interestingly, there was a distinctive difference in segregation in these dyads. The predominant class in *dfp1-r35* mutants came from asci that underwent a single reductional division (55.57% supplementary material Table S6 class A). However, in *rec12*Δ mutants, the predominant dyads two meiotic divisions, but formed a single spore around two nuclear bodies, resulting in an apparent bi-nuclear dyad (44.68% supplementary material Table S6 class B).

### Rec8 dynamics in Dfp1 C-terminal truncation mutants

The differences in chromosome segregation defects in *dfp1-r35* and *rec12*Δ are consistent with data from budding yeast suggesting that DDK is required to phosphorylate and inactivate the Rec8 cohesin that maintains association between sister chromatids (Green et al., 2009; Lo et al., 2008; Matos et al., 2008; Molnar et al., 1995; Parisi et al., 1999; Rabitsch et al., 2003; Tóth et al., 2000; Watanabe and Nurse, 1999). Deletion of *S. cerevisiae rec8* partly rescues the phenotype associated with a *cdc7* mutant (Valentin et al., 2006). We constructed homozygous double mutant diploids *dfp1-r35 rec12*Δ or *dfp1-r35 rec8*Δ. Both of these partly rescued the *dfp1-r35* dyad phenotype from 75% to 44.44% and 44.83% respectively (Fig. 5; supplementary material Table S5). The *dfp1-r35 rec8*Δ double mutant showed the same fraction of equational MI divisions as the *rec8*Δ single mutant, indicating that *rec8*Δ is epistatic to *dfp1-r35* (Fig. 5; supplementary material Table S5).

The *dfp1-r35* phenotype resembles the phenotype observed in mutants containing a non-cleavable form of Rec8 (Kitajima et al., 2003), and the partial rescue of *dfp1-r35* dyad formation by *rec8*Δ suggests that some of its defect reflects the inability to inactivate Rec8. We therefore examined whether Rec8 stability is affected by deletion of the C-terminus of Dfp1, using live cell imaging and western blot analysis in wild type and mutant cells.

**Fig. 6.**
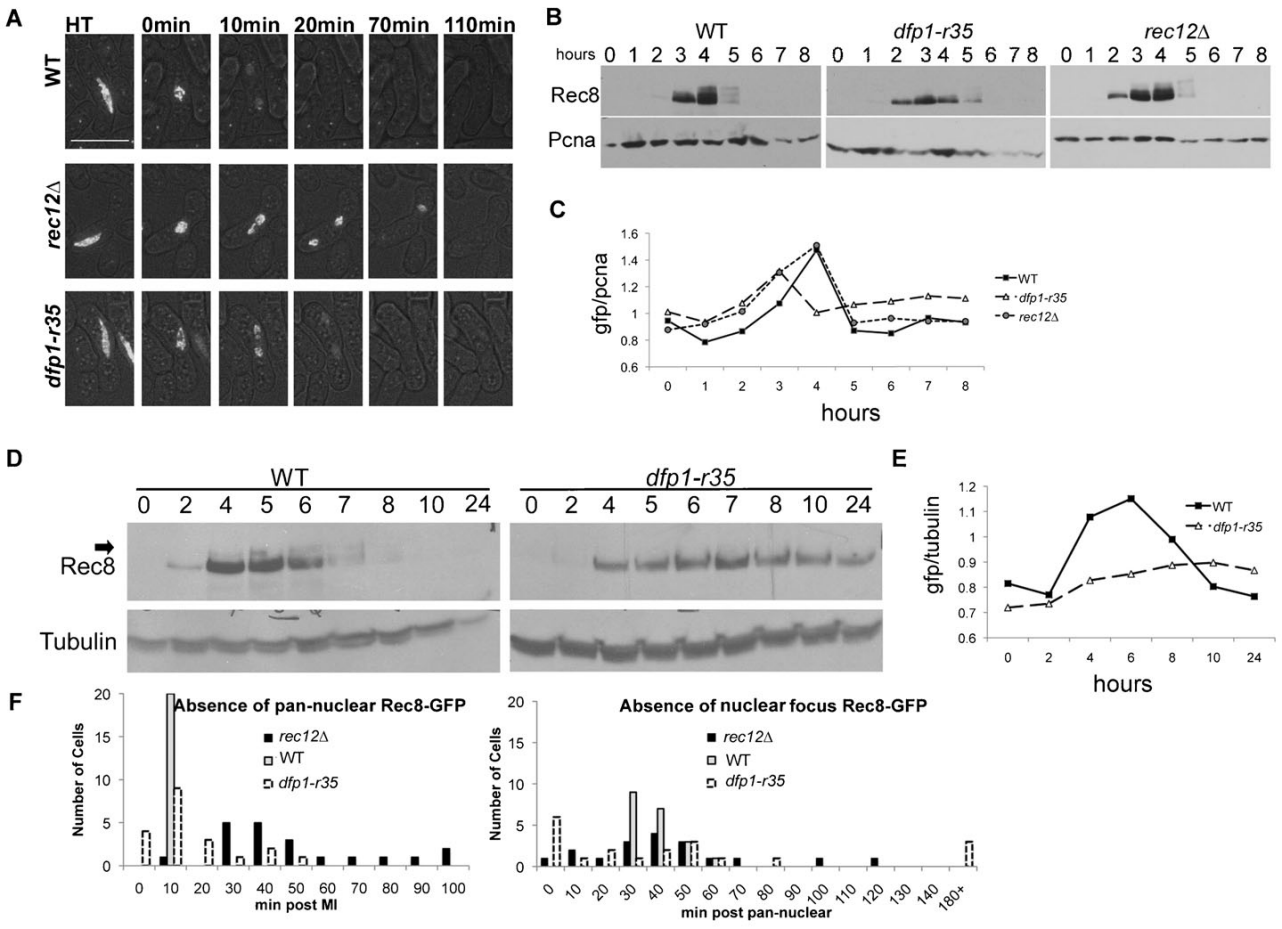
Rec8-GFP stability. (**A**) Representative images from live cell imaging observing diploid meiosis in cells containing Rec8-GFP (supplementary material Movies 7–9). The indicated strains were grown at (32°C temperature) and plated on agarose pads for image acquisition (see Materials and Methods). Strains: wild type (FY6173×FY6174), *dfp1-r35* (FY6241×FY6242), *rec12Δ* (FY6217×6218). Scale bar: 10 µm. (**B**) Western blot of Rec8-GFP during synchronous meiosis in *h^−^/mat2-102 pat1-114*/*pat1-114* stable diploid. Strains: wild type (FY6332×FY6336), *dfp1-r35* (FY6347×FY6378) *rec12*Δ (FY6530×FY6531). (**C**) Quantification of panel B using GFP signal/PCNA. (**D**) Western blot of Rec8-GFP in haploid *pat1-114* meiosis. Strains: wild type (FY4129) and *dfp1-r35* (FY4396). (**E**) Quantification of panel D as done in panel C. (**F**) Graphical representation of quantification of Rec8-GFP timing in live cell imaging in panel A. Pan-nuclear signal disappearance occurred at an average of 10 mins, 15.5 mins, and 50.5 mins post MI for wild type, *dfp1-r35*, and *rec12*Δ respectively with *P*-values using a two-tailed t-test of 0.0000008 and 0.102 for *rec12*Δ and *dfp1-r35* respectively. For the disappearance of the nuclear focus relative to the disappearance of the pan-nuclear signal the timing was an average of 48 mins, 56.6 mins, and 55.5 mins for wild type, *dfp1-r35*, and *rec12*Δ with *P*-values using a two-tailed t-test of 0.258 and 0.599 for *rec12*Δ and *dfp1-r35* respectively.

As described previously (Watanabe and Nurse, 1999), we observed that wild type diploids accumulate a pan-nuclear signal of rec8-GFP during meiosis. This signal is reduced to two puncta at the MI division, which completely disappear following MII (Fig. 6A; supplementary material Movies 7–9). We noted a slight delay in the disappearance of Rec8-GFP in *dfp1-r35* cells relative to the MI division. In wild type the pan-nuclear signal decreased to a single focus at 10 min post MI; however, *dfp1-r35* showed this with an average timing of 15.5 min. Also, the *dfp1-r35* cells had a large variation in the timing, while the wild type cells behaved more consistently (Fig. 6F). Surprisingly, we saw an even more pronounced delay in *rec12*Δ mutants, in which the pan-nuclear signal persisted for an extended time of an average of 50.5 mins. Again we observed a larger variation in the mutant compared to wild type; however, the difference between wild type and *rec12*Δ is significant using a two-tailed t-test with a *P*-value of 8×10^−7^ (Fig. 6F). In wild type, Rec8-GFP foci disappeared 48 mins after the conversion from pan-nuclear signal to single focus; while this disappearance occurred at 56.5 mins in *dfp1-r35*, and 55.5 min in *rec12Δ* (Fig. 6F).

In order to get a higher resolution analysis of Rec8-GFP dynamics in both *dfp1-r35* and *rec12*Δ mutants, we induced synchronous meiosis in a *mat2-102* stable diploid using *pat1-114* temperature inactivation, and used western blots to visualize protein levels (Fig. 6B,C). In wild type cells, Rec8-GFP protein levels peak at four hours and are undetectable by 6 hours (Fig. 6B,C). We observed an electrophoretic mobility shift in Rec8-GFP at four and five hours in wild type cells, prior to its disappearance. This corresponds to a previously reported phospho-shift found to be important for Rec8 degradation (Ishiguro et al., 2010; Parisi et al., 1999; Rumpf et al., 2010). We observed that the amount of Rec8-GFP in *dfp1* mutants levels did not increase to the level of wild type. The peak of Rec8-GFP in *dfp1-r35* was observed at three hours rather than four as in wild type, and a low level of Rec8-GFP was still detectable in the mutant at later time points. The Rec8 mobility shift was undetectable in *dfp1* mutant. *rec12*Δ diploids were similar to wild type in terms of Rec8-GFP dynamics and mobility shift. Thus, the prolonged pan-nuclear staining we observe during MI is not due to changes in protein level, but to defects in localization.

We observed that the *dfp1-r35* Rec8-GFP phenotype is more pronounced in haploids compared to diploids (Fig. 6D,E). There was a reduced level of total protein, and Rec8-GFP persisted even after 24 hours post meiotic induction, suggesting that the inability of haploid *pat1 dfp1-r35* mutants to undergo a second meiotic division is due to the persistence of Rec8 in this strain. Interestingly, the mobility shift associated with phosphorylation was much less apparent in the haploid than the diploid.

## Discussion

DDK was first identified as an S phase specific kinase essential for DNA replication (Duncker and Brown, 2003; Kim et al., 2003; Labib, 2010; Sclafani, 2000). Subsequent studies have linked it to a wide range of activities temporally linked to S phase. Separation of function alleles in the fission yeast DDK regulatory subunit *dfp1^+^* suggest that different functions may map to different Dfp1 domains (Bailis et al., 2003; Dolan et al., 2010; Fung et al., 2002; Hayashi et al., 2009). Previously, the Dfp1 C-terminus was shown to be important to the response to alkylation damage, possibly through mediating trans-lesion synthesis repair (Dolan et al., 2010; Fung et al., 2002). Our current work shows that the C-terminus of Dfp1, which contains in a zinc finger domain truncated by the *dfp1-r35* mutation, influences DDK activity during meiosis, leading to multiple defects including loss of programmed double strand breaks, disruption in the meiotic transcription program, and defects in turnover of the meiotic cohesin Rec8.

Early studies on DDK in meiosis in *S. cerevisiae* linked DDK to meiotic recombination, rather than replication (Buck et al., 1991; Sclafani et al., 1988). Subsequently, it was determined that DDK is important for activation of the Rec12/Spo11 endonuclease in both budding and fission yeasts (Ogino and Masai, 2006; Sasanuma et al., 2008; Wan et al., 2008; Wan et al., 2006). In budding yeast, DDK phosphorylates Mer2 (Sasanuma et al., 2008; Wan et al., 2008), although a homologue of this substrate has not been identified in fission yeast. We show that *S. pombe dfp1-r35* mutants are proficient for meiotic S phase, but defective in prDSB formation, with dramatically reduced recombination. The residual recombination in *dfp1-r35* relative to *rec12*Δ may reflect the presence of a low level of DNA damage and double strand breaks in *dfp1-r35* (Dolan et al., 2010); there is evidence that mutations that cause low levels of DNA damage may provide Rec12-independent substrates for recombination (Farah et al., 2005a; Farah et al., 2005b; Pankratz and Forsburg, 2005).

The defects of *dfp1-r35* in meiosis are far more severe than those observed in *rec12*Δ cells, including substantially reduced spore viability and disruptions of segregation, indicating it plays roles beyond induction of programmed double strand breaks. Consistent with this, we also observed dysregulation of meiosis-specific gene expression in *dfp1* mutants. Early transcripts (including *rec8*^+^, *rec12*^+^, and *dfp1*^+^ itself) accumulate with normal timing. However, accumulation of mid-meiotic transcripts, including *mei4^+^*, is noticeably delayed in *dfp1-r35* mutants. Mei4 is a fork-head transcription factor that is responsible for expression of numerous downstream genes required for meiotic divisions and sporulation (Horie et al., 1998; Mata et al., 2002). Expression of *mei4*^+^ occurs early in meiosis, but there is no accumulation of its transcript due to regulated turnover by the Mmi1 protein, operating through the DSR sequence element (Harigaya et al., 2006). The same Mmi1 system contributes to the turnover of early meiotic transcripts such as *rec8*^+^ (Yamanaka et al., 2010). Because the *rec8*^+^ transcript accumulates normally in *dfp1-r35* cells, the failure to accumulate *mei4*^+^ cannot be due to a failure to inactivate Mmi1.

There is evidence in budding yeast that its mid-meiosis transcription factor, Ndt80, is regulated by DDK through an indirect mechanism (Lo et al., 2012). Although ScNdt80 performs a similar function to Sp Mei4, the proteins are not structurally related. It is possible that the DDK regulation of the mid-meiotic transcription factors occurs through other, more conserved proteins. Alternatively, there may be broad functional similarities but molecularly distinct mechanisms involved.

We also find that *dfp1* cells largely bypass the MII division, forming dyads. Loss of viability and dyad formation can be partly rescued by deletion of Rec8 cohesin, suggesting that persistence of Rec8 in *dfp1* hinders meiotic progression. Previously, we showed that Dfp1 interacts with Psc3, a component of the mitotic cohesin complex (Bailis et al., 2003), which could suggest a direct interaction with the cohesins. We find that the characteristic Rec8 phosphorylation mobility shift is reduced in *dfp1* cells, and the Rec8 protein persists until late in meiosis. This is particularly apparent in haploid cells, and suggests that Rec8 turnover may be DDK-dependent. Turnover of Rec8 is driven by phosphorylation that depends in part upon casein kinase 1 (Ishiguro et al., 2010; Parisi et al., 1999; Rumpf et al., 2010; Katis et al., 2010). Our study indicates that Rec8 phosphorylation also depends upon DDK. This is consistent with work from budding yeast suggesting that DDK collaborates with CK1 in Rec8 phosphorylation (Katis et al., 2010). *A priori*, we cannot conclude whether DDK acts directly upon Rec8, or whether it regulates the activity of CK1, although based on analogy to budding yeast, the former seems likely.

Together, these observations link DDK to multiple functions in fission yeast meiosis as in budding yeast: induction of programmed DSBs, regulation of the mid-meiotic transcription program, and turnover of the Rec8 cohesin required for proper MII division. This is particular striking as the overall logic of meiotic regulation, and many drivers of meiotic progression, are not conserved in the two yeasts.

How does one domain of Dfp1 target the DDK to such diverse activities? One possibility is that this mutation simply reduces kinase activity, as proposed by Fung et al. and Harkins et al. (Fung et al., 2002; Harkins et al., 2009). However, *dfp1-r35* cells are competent for replication in both vegetative and meiotic cells, which shows that that sufficient kinase activity remains for this essential function, even though meiosis is severely disrupted. If a loss of kinase activity is responsible for all the defects we observe, it would suggest a substantial difference in the threshold kinase activity required for different activities. Another possibility is that the C-terminal zinc finger domain truncated in *dfp1-r35* contributes to substrate recognition. We showed previously that Dfp1 chromatin association in response to MMS treatment is disrupted by loss of this domain (Dolan et al., 2010). Since meiotic cohesion and prDSB formation are both linked to progression of S phase (Doll et al., 2008; Kugou et al., 2009; Ogino and Masai, 2006), it is plausible that both Rec8 and Rec12 meet the replication fork, and possibly, a fork-linked DDK.

An additional finding of our study is a significant difference between the phenotype of *dfp1-r35* mutants in haploid and diploid driven meiosis, with the haploid being more severely affected. These differences are particularly relevant because the *pat1* temperature-induction system in haploids is frequently used to model meiosis in fission yeast. Previously, it was shown that monopolar spindle attachment prior to the MI division, which is essential for reductional segregation, has different requirements depending on the ploidy of the cell (Yamamoto and Hiraoka, 2003). In haploids, monopolar attachment depends on Pat1 inactivation, pheromone signaling, and Rec8. However, it is independent of Rec12 and recombination. In contrast, in diploids, monopolar attachment is independent of pheromone signaling but requires Rec12 and recombination, in addition to Rec8 and Pat1 inactivation.

In our study, we observe that haploid *pat1-114 dfp1-r35* cells fail to proceed through MII of meiosis, accompanied by failure to degrade the Rec8 cohesin. Interestingly, we see is a more modest mobility shift of Rec8 in wild type haploids compared to diploids. We speculate that this might indicate that the role of CK1 in Rec8 phosphorylation is reduced in haploids, although this remains to be tested.

In contrast, in diploid cells homozygous for *pat1-114 dfp1-r35*, even though the majority of cells do not complete MII, we see only a modest reduction of Rec8 turnover; a similar phenotype is observed for a *pat1^+^ dfp1-r35* diploid. The MII arrest is partly (but not completely) rescued by *rec8*Δ, indicating that diploids homozygous for *dfp1-r35* have additional defects beyond difficulties in regulating Rec8. These likely include disruptions in recombination and the transcriptional program, as we observe, or changes in chromosome or rDNA segregation as reported by Bailis and Forsburg, and Sullivan et al. (Bailis and Forsburg, 2004; Sullivan et al., 2008).

By analogy to the effect on monopolar spindle attachment reported by Matos et al. and Yamamoto and Hiraoka (Matos et al., 2011; Yamamoto and Hiraoka, 2003), our data may also suggest that a Rec12-dependent pathway in the diploid ameliorates the effects due to *dfp1-r35* just as it contributes to monopolar attachment. Interestingly, Rec12 protein persists and has been suggested to play a role in MII (DeWall et al., 2005), so there may be some additional effects of proper pairing of homologous chromosomes in the context of recombination that is independent of DDK. These results provide a caveat in the use of haploid *pat1* strains to model meiotic progression.

## Supplementary Material

Supplementary Material
