# Supplementary material for: The C-terminus of S. pombe DDK subunit Dfp1 is required for meiosis-specific transcription and cohesin cleavage
Source: Biol Open. 2013 Jun 11;2(7):728–38. doi: 10.1242/bio.20135173 (PMC3711041; doi:10.1242/bio.20135173)
Supplement: Supplementary Material [file supp_2_7_728__index.html]

The C-terminus of S. pombe DDK subunit Dfp1 is required for meiosis-specific transcription and cohesin cleavage — The C-terminus of S. pombe DDK subunit Dfp1 is required for meiosis-specific transcription and cohesin cleavage — Supplementary Material 

# The C-terminus of *S. pombe* DDK subunit Dfp1 is required for meiosis-specific transcription and cohesin cleavage

## 

**Files in this Data Supplement:**

- Supplementary Material - Anh-Huy Le et al. doi: 10.1242/bio.20135173
- Movie 1 - **Movie 1. Representative movie of live cell imaging.** Supplementary material Movies 1–6 are the primary data for the time-lapse images in Fig. 5. Yellow signal is LacI-GFP. Image is fusion of transmitted light and GFP signal. This movie shows the following genotype: wild type. Scale bar: 10 µm.
- Movie 2 - **Movie 2. Representative movie of live cell imaging.** This movie shows the following genotype: *dfp1-r35*. Scale bar: 10 µm.
- Movie 3 - **Movie 3. Representative movie of live cell imaging.** This movie shows the following genotype: *rec12*Δ. Sca0le bar: 10 µm.
- Movie 4 - **Movie 4. Representative movie of live cell imaging.** This movie shows the following genotype: *rec8*Δ. Scale bar: 10 µm.
- Movie 5 - **Movie 5. Representative movie of live cell imaging.** This movie shows the following genotype: *dfp1-r35 rec12*Δ. Scale bar: 10 µm.
- Movie 6 - **Movie 6. Representative movie of live cell imaging.** This movie shows the following genotype: Δ, *dfp1-r35 rec8Δ*. Scale bar: 10 µm.
- Movie 7 - **Movie 7. Representative movie of live cell imaging of Rec8-GFP in asynchronous meiosis.** Supplementary material Movies 7–9 are the primary data for the time-lapse images in Fig. 6. Green signal is Rec8-GFP. Image is fusion of transmitted light and GFP signal. This movie shows the following genotype: wild type. Scale bar: 10 µm.
- Movie 8 - **Movie 8. Representative movie of live cell imaging of Rec8-GFP in asynchronous meiosis.** This movie shows the following genotype: *dfp1-r35*. Scale bar: 10 µm.
- Movie 9 - **Movie 9. Representative movie of live cell imaging of Rec8-GFP in asynchronous meiosis.** This movie shows the following genotype: *rec12* Δ. Scale bar: 10 µm.
