# Supplementary material for: The C-terminus of S. pombe DDK subunit Dfp1 is required for meiosis-specific transcription and cohesin cleavage
Source: Biol Open. 2013 Jun 11;2(7):728–38. doi: 10.1242/bio.20135173 (PMC3711041; doi:10.1242/bio.20135173)
Supplement: Supplementary Material [file supp_bio.20135173_bio.20135173-s1.pdf]

Supplementary Material

Anh-Huy Le et al. doi: 10.1242/bio.20135173

Table S1. Strains used.

Name	Genotype	Reference
FY155	*h90 968*	Our stock
FY1154	*h90 dfp1-r35 ura4-D18 leu1-32*	Henning Schmidt
FY1319	h^{-} *Δrec8::ura4*$^{+}$ *leu1-32 ura4-D18 ade6-M210*	Our stock
FY2008	h^{-} *pat1-114 Δrec12::LEU2 leu1-32 ade6-M216*	Our stock
FY3973	h^{-} *dfp1-r35 leu1-32 ura4-D18 ade6-M210*	This work
FY4129	h^{-} *pat1-114 ura4-D18 ade6-M216*	This work
FY4396	h^{-} *pat1-114 dfp1-r35 ade6-M216*	This work
FY4397	h^{-} *dfp1-r35 ura4-D18 ade6-M52 lys4-95*	This work
FY4398	h^{-} *ura4-D18 ade6-M52 lys4-95*	This work
FY4405	h^{+} *leu1-32 ade6-M26 his4-239*	This work
FY4518	h^{+} *dfp1-r35 leu1-32 ade6-M26 his4-239*	This work
FY4561	h^{+} *Δrec12::LEU2 ura4-D18 leu1-32 ade6-M26 his4-239*	This work
FY4843	h^{+} *dfp1-r35 sad1*$^{+}$*::DsRed-LEU2 his7*$^{+}$*::lacI-GFP lys1*$^{+}$*::lacO leu1-32 ura4-D18 ade6-M216*	This work
FY4915	h^{+} *Δrec8::ura4*$^{+}$ *sad1*$^{+}$*::DsRed-LEU2 his7*$^{+}$*::lacI-GFP lys1*$^{+}$*::lacO leu1-32 ura4-D18 ade6-M216*	This work
FY4991	h^{-} *pat1-114 rec8GFP::Kan leu1-32 ade6-M210 can1-1*	This work
FY5164	h^{-} *pat1-114 dfp1-r35 rec8GFP::Kan leu1-32 ade6-M210*	This work
FY5268	h^{-} *Δrec12::ura4*$^{+}$ *ura4-D18 leu1-32 ade6-M52 lys4-95*	This work
FY5916	h^{+} *Δrec8::ura4*$^{+}$ *his7*$^{+}$*::lacI-GFP lys1*$^{+}$*::lacO ura4-D18 leu1-32*	This work
FY5917	h^{+} *Δrec12::ura4*$^{+}$ *his7*$^{+}$*::lacI-GFP lys1*$^{+}$*::lacO leu1-32 ura4-D18*	This work
FY6131	h^{+} *leu1-32 ura4-D18 rec8::ura4*$^{+}$ *arg3*$^{+}$*::D0817-mCherry::his5*$^{+}$ *his5D?*	This work
FY6134	h^{-} *ura4-D18 rec12::ura4*$^{+}$ *arg3*$^{+}$*::D0817-mCherry::his5*$^{+}$ *leu1-32 hsi5D?*	This work
FY6143	h^{-} *Δrad35-271 rec12::ura4*$^{+}$ *his7*$^{+}$*::lacI-GFP lys1*$^{+}$*::lacO leu1-32 ura4-D18*	This work
FY6173	h^{+} *rec8-GFP-kan leu1-32 ade6-M210*	This work
FY6174	h^{-} *rec8-GFP-kan leu1-32 ade6-M210*	This work
FY6175	h^{-} *ura4-D18 rad35-271 rec12::ura4*$^{+}$ *arg3*$^{+}$*::D0817-mCherry::his5*$^{+}$ *leu1-32 hsi5D?*	This work
FY6204	h^{-} *rad35-271 Δrec8::ura4*$^{+}$ *arg3*$^{+}$*::D0817-mCherry::his5*$^{+}$ *his5D? ura4-D18 leu-32*	This work
FY6217	h^{+} *rec8-GFP-kan Δ rec12::ura4*$^{+}$ *ade6-M210 leu1-32 ura4-D18*	This work
FY6218	h^{-} *rec8-GFP-kan Δrec12::ura4*$^{+}$ *ade6-M210 leu1-32 ura4-D18*	This work
FY6221	h^{+} *his7*$^{+}$*::lacI-GFP lys1*$^{+}$*::lacO leu1-32*	This work
FY6236	h^{+} *rad35-271 arg3*$^{+}$*::D0817-mCherry::his5*$^{+}$ *his5D? leu1-32*	This work
FY6241	h^{-} *rad35-271 rec8-GFP-kan leu1-32*	This work
FY6242	h^{+} *rad35-271 rec8-GFP-kan leu1-32*	This work
FY6331	h^{-} *arg3*$^{+}$*::D0817-mCherry::his5*$^{+}$ *leu1-32 his5D*	This work
FY6332	*h90 mat2-102 pat1-114 rec8-GFP-kan ade6-M210*	This work
FY6336	h^{-} *pat1-114 rec8-GFP-kan ade6-M216*	This work
FY6347	h^{-} *pat1-114 rad35-271 rec8-GFP-kan ade6-M216*	This work
FY6416	h^{+} *rad35-271 Δrec8::ura4*$^{+}$ *his7*$^{+}$*::lacI-GFP lys1*$^{+}$*::lacO ura4-D18 leu1-32*	This work
FY6441	h^{+} *rad35-271 his7*$^{+}$*::lacI-GFP lys1*$^{+}$*::lacO leu1-32*	This work
FY6478	*h90 mat2-102 pat1-114 rad35-271 rec8-GFP-kan leu1-32 ade6-M210*	This work
FY6530	*h90 mat2-102 pat1-114 rec12Δ::ura4*$^{+}$ *ura4-D18 rec8-GFP-kan ade6-M210*	This work
FY6531	h^{-} *pat1-114 rec12Δ::ura4*$^{+}$ *ura4-D18 rec8-GFP-kan ade6-M216*	This work

Table S2. Oligos used for RT-PCR.

Target	Oligo sequence (5′-3′)	Source
mei4	TTGGAGATGAAATGGCGGGCTTTG	This work
mei4	GTAGCGAAACGTGTTGCGAATCCA	This work
cdc25	GCACAGAGCACCTCATTTGGCATT	This work
cdc25	AACATGCGAAGCATCGTTCATCGG	This work
mde10	CAGTTCGCTTTGCGATGCTCAAGA	This work
mde10	AACGATGCCGTTTCCACATGTTCC	This work
rec25	GAGAGAGACACCGGACATGAT	This work
rec25	CCCTTGTATAAGCTGAGACCCTTG	This work
psm3	TTCAAGGTCGCTGTTGAGGCTACT	This work
psm3	TGGCATCAGGATAAGTAACCGCCT	This work
rec12	CCGAAGAGGCTTAGCGGATACAA	This work
rec12	ACTGTGCGGTTCATGACTGTAGG	This work
act1	TGCACCTGCCTTTTATGTTG	(Kloc et al., 2008)
act1	TGGGAACAGTGTGGGTAACA	(Kloc et al., 2008)

Biology Open

Table S3. Spore viability. Fixed numbers of spores were plated after counting. The standard deviation was calculated from four trials of the experiment.

	Total plated	Total CFU	% Viable spores		Std dev.	Fold reduction
			Absolute	Normalized		
WT	19000	11081	58.32	100.00	15.72	
rec12Δ	100000	12240	12.24	20.99	5.17	4.76
dfp1-r35	400000	4225	1.06	1.81	1.46	55.22

Table S4. Recombination rates. Comparison of intergenic recombination rates between *his4-239* and *lys4-95* on Chromosome II and intragenic recombination at *ade6-M26* and *ade6-52*. Data are pooled from four trials.

	Total	# His^+ Lys^+ (diploid)	# His^+ Lys^+ (haploid)	# Ade^+	% His^+ Lys^+	% Ade^+
WT	8758	0	341	25	3.985	0.285
rec12Δ	9950	142	1	0	0.010	<0.010
dfp1-r35	13892	88	15	1	0.108	0.007

Table S5. Summary of classes from supplementary material Table S6, as a percent of the total.

	WT	*dfp1-r35*	*rec12Δ*	*rec8Δ*	*dfp1-r35 rec12Δ*	*dfp1-r35 rec8Δ*
1 spore (J)	0.00	0	0	0	0	10.34
2 spores (A,C,D,E,I)	2.56	75.00	42.55	0.00	44.44	44.83
3 spores (F)	0.00	2.78	10.64	0.00	0.00	0.00
4 spores (B,G,H)	97.44	22.22	46.81	100.00	55.56	44.83
1st is reductional (A,B,C,F,G,I)	92.31	80.56	95.74	8.33	55.56	10.34
1st is equational (D,E,H,J)	7.69	19.44	4.26	91.67	44.44	89.66

Table S6. Chromosome segregation monitored with LacI-GFP. Chromosome segregation assay using live cell imaging and parental strains heterozygous for LacO array. Cells were sorted into different segregation classes dependent on whether a reductional or equational division occurred at MI and how many spores were formed in the terminal product. Data are pooled from at least 3 biological replicates.

Class definitions					Percentage of classes					
Class	# Divisions	1st division	2nd division	# Spores	WT	*dfp1-r35*	*rec12Δ*	*rec8Δ*	*dfp1-r35 rec12Δ*	*dfp1-r35 rec8Δ*
A	1	R	no	2	0.00	55.56	6.38	0.00	22.22	0.00
B	2	R	E	4	89.74	16.67	44.68	8.33	33.33	6.90
C	2	R	E	2	2.56	2.78	29.79	0.00	0.00	0.00
D	2	E	yes	2	0.00	2.78	0.00	0.00	0.00	0.00
E	1	E	no	2	0.00	13.89	4.26	0.00	22.22	41.38
F	2	R	E	3	0.00	2.78	10.64	0.00	0.00	0.00
G	2	R	R	4	0.00	2.78	2.13	0.00	0.00	0.00
H	2	E	yes	4	7.69	2.78	0.00	91.67	22.22	37.93
I	2	R	R	2	0.00	0.00	2.13	0.00	0.00	3.45
J	1	E	no	1	0.00	0.00	0.00	0.00	0.00	10.34
Total cells analyzed					39	36	47	36	27	29

E=equational division; R=reductional division.

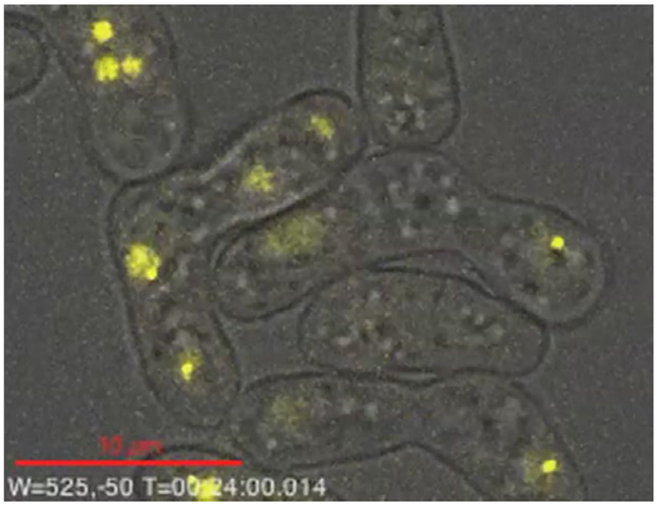

Movie 1. Representative movie of live cell imaging. Supplementary material Movies 1–6 are the primary data for the time-lapse images in Fig. 5. Yellow signal is LacI-GFP. Image is fusion of transmitted light and GFP signal. This movie shows the following genotype: wild type. Scale bar: 10 µm.

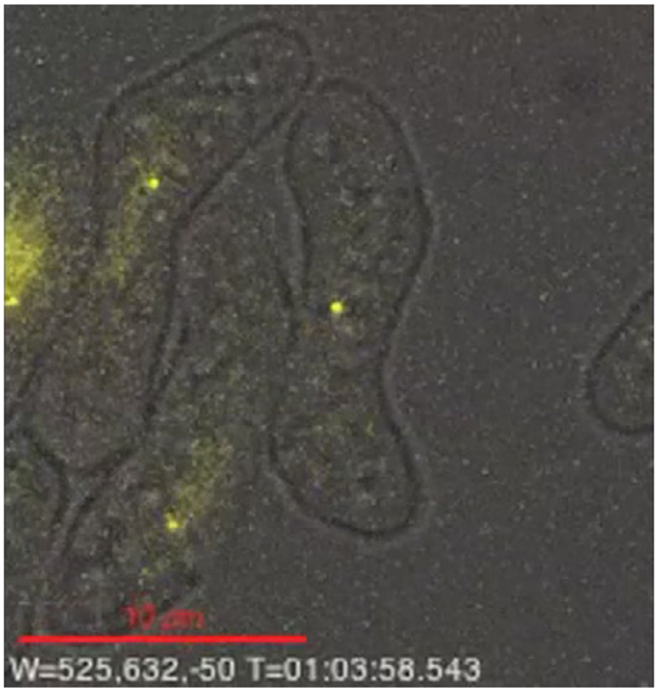

Movie 2. Representative movie of live cell imaging. This movie shows the following genotype: *dfp1-r35*. Scale bar: 10 µm.

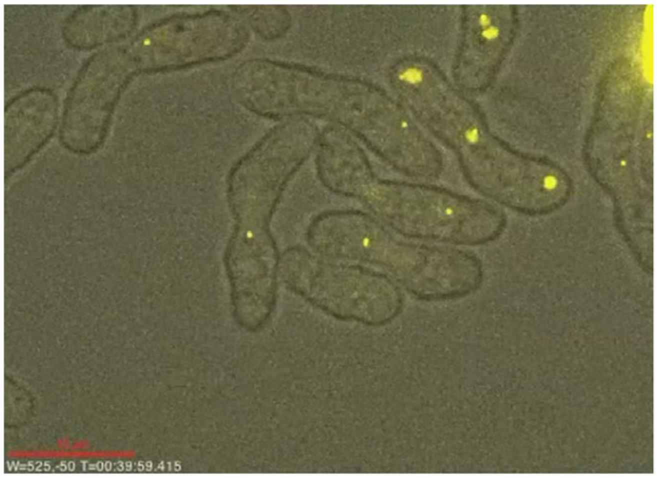

Movie 3. Representative movie of live cell imaging. This movie shows the following genotype: *rec12*Δ. Sca0le bar: 10 µm.

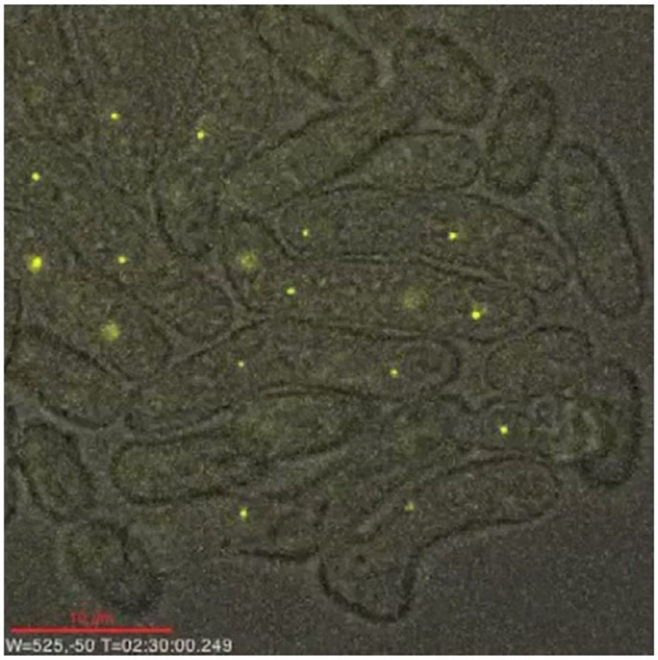

Movie 4. Representative movie of live cell imaging. This movie shows the following genotype: *rec8*Δ. Scale bar: 10 µm.

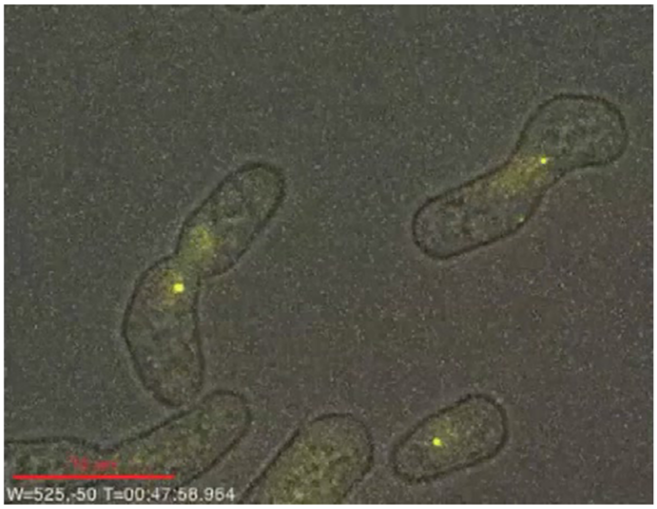

Movie 5. Representative movie of live cell imaging. This movie shows the following genotype: *dfp1-r35 rec12Δ*. Scale bar: 10 µm.

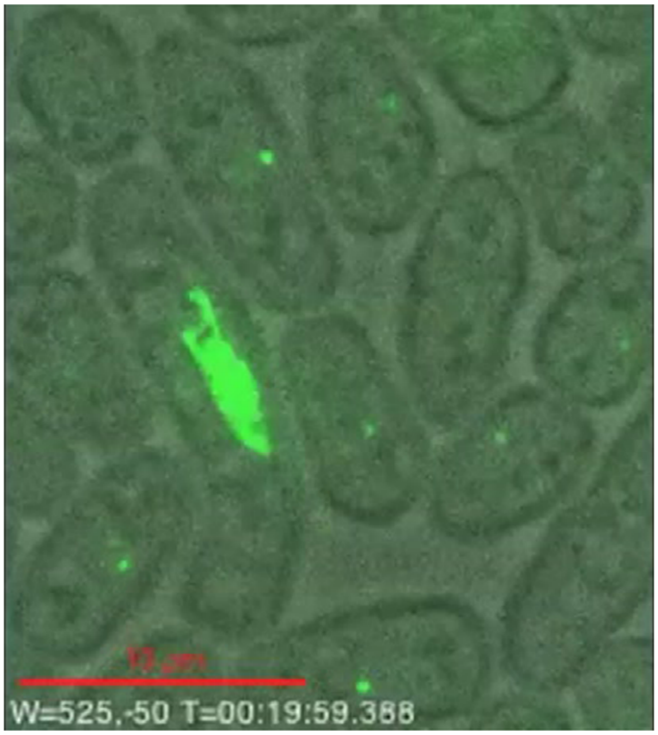

Movie 7. Representative movie of live cell imaging of Rec8-GFP in asynchronous meiosis. Supplementary material Movies 7–9 are the primary data for the time-lapse images in Fig. 6. Green signal is Rec8-GFP. Image is fusion of transmitted light and GFP signal. This movie shows the following genotype: wild type. Scale bar: 10 µm.

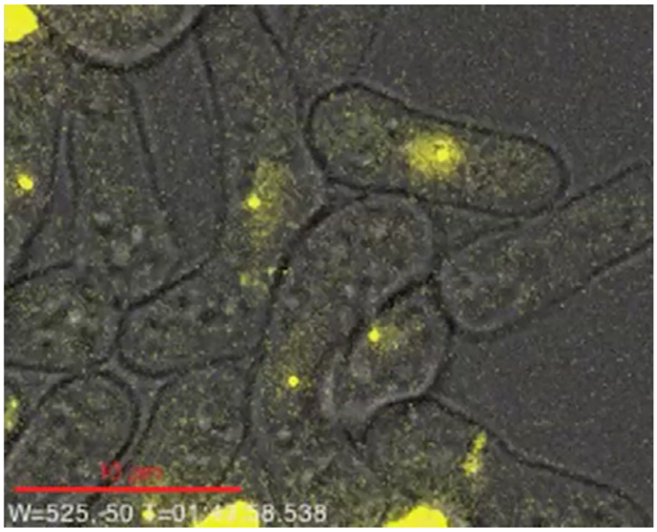

Movie 6. Representative movie of live cell imaging. This movie shows the following genotype: Δ, *dfp1-r35 rec8Δ*. Scale bar: 10 µm.

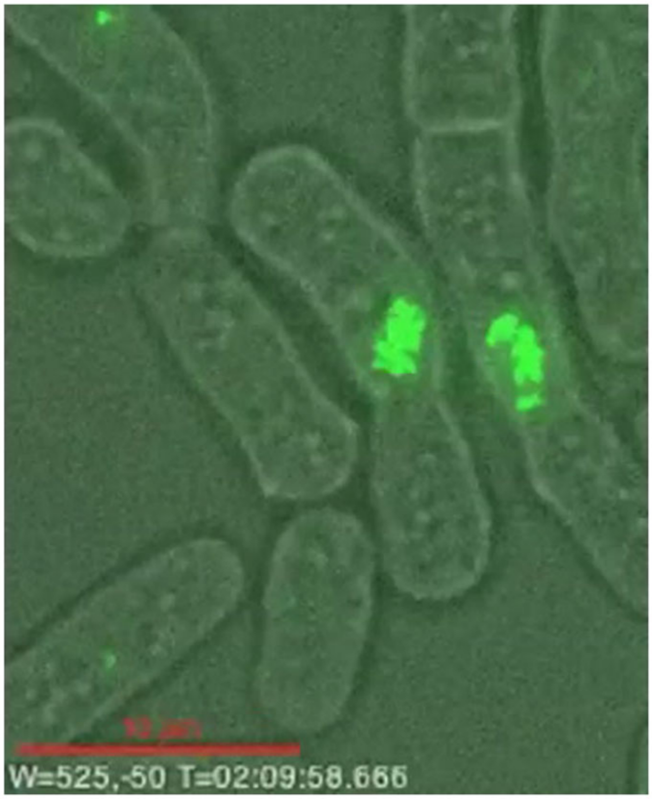

Movie 8. Representative movie of live cell imaging of Rec8-GFP in asynchronous meiosis. This movie shows the following genotype: *dfp1-r35*. Scale bar: 10 µm.

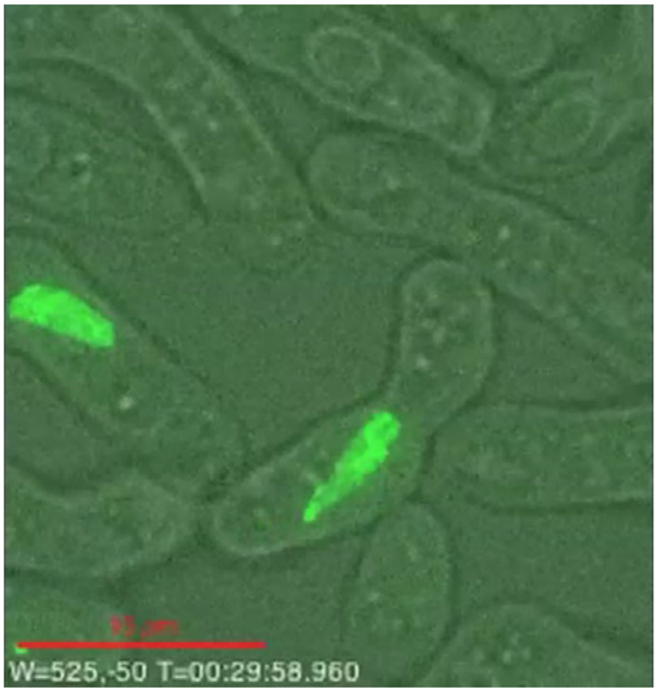

Movie 9. Representative movie of live cell imaging of Rec8-GFP in asynchronous meiosis. This movie shows the following genotype: *rec12* Δ. Scale bar: 10 µm.
